# Knowledge base and online identification key of native Asian thorny bamboos within *Bambusa* (Bambusoideae, Poaceae)

**DOI:** 10.3897/phytokeys.270.171208

**Published:** 2026-02-03

**Authors:** Khac Dieu Nguyen, My Hanh Diep, Marc Pignal, Régine Vignes Lebbe

**Affiliations:** 1 Viet Nam National University, Ho Chi Minh City, 700000, Vietnam Phu An Bamboo Village, University of Science Ho Chi Minh City Vietnam; 2 Phu An Bamboo Village, University of Science, Ho Chi Minh City, Vietnam Viet Nam National University Ho Chi Minh City Vietnam; 3 Institut de Systématique, Evolution, Biodiversité (ISYEB), Muséum national d'Histoire naturelle, CNRS, Sorbonne Université, EPHE, Université des Antilles, 75005 Paris, France Université des Antilles Paris France

**Keywords:** Asia, *
Bambusa
*, online identification key, Poaceae, thorny bamboos, Xper^3^

## Abstract

This study presents a checklist of 13 species of thorny bamboos native to Asia and documents them to contribute to a broader understanding of their morphology. To support this list and facilitate species recognition, a morphological database was compiled from a wide range of published sources and paired with an interactive identification key. This key incorporates diagnostic illustrations, descriptive characters, and precise definitions, thereby enabling more accurate identification. In addition to morphological traits, the database includes general notes, taxonomic synonymy, and bibliographic references for each species. Initial testing was conducted with specimens housed at the MNHN Herbarium in Paris, representing a first step toward validation. The current version provides a useful reference for both field and herbarium studies, and the framework has been designed for continuous updating as new data become available. Detailed results of the identification key are available at https://thornybamboo_asia.identificationkey.org.

## Introduction

Bamboos are giant grasses that make up the subfamily Bambusoideae Luerss. (Poaceae (R.Br.) Barnh.). There are 136 genera within Bambusoideae, with about 1,698 species worldwide, arranged into three tribes: Arundinarieae Asch. & Graebn., Bambuseae Kunth ex Dumort., and Olyreae Kunth ex Spenn. (Vorontsova et al. 2016; Soreng et al. 2022). They are naturally distributed on all continents except Europe (Dransfield and Widjaja 1995).

*Bambusa* (Bambusoideae, Poaceae), the type genus of the subtribe Bambusinae J.Presl, is a large and complex genus distributed in tropical and subtropical monsoon climates, with around 150 species worldwide. Of these, about 80 species occur in China and 60–70 species in Vietnam—two countries contributing nearly 50% of the total species in this genus. Other countries in this region, such as Thailand and India, have recorded 10–15 species and about 25 species, respectively (Seethalakshmi et al. 1998; Ohrnberger 1999; Xia et al. 2006; Wong and Diep 2015; Arthan et al. 2023). *Bambusa* species are typically characterized by their conspicuous, broad, auricle-like lobes on the culm sheath (Wong 1993; Xia et al. 2006), in contrast to the absence or inconspicuous presence of culm sheath auricles in species of *Gigantochloa* Kurz ex Munro and *Dendrocalamus* Nees (Li and Stapleton 2006). However, auricle characteristics similar to those of the two genera mentioned above are also observed in some *Bambusa* species, such as *B. flexuosa* Munro.

The type species of *Bambusa* is *Arundo bambos* L. (now *B. bambos* (L.) Voss), a thorny bamboo widely distributed from the Indian subcontinent to Indochina (Wong 1993; Xia and Stapleton 1997; POWO 2025). The designation “thorny bamboo” is generally applied to a category of large-sized *Bambusa* Schreb. species with thorn-like structures on the lower part of the culms (Pham 1993; Dransfield and Widjaja 1995; Nguyen 2006). Some common species can reach up to 30 m tall and 20 cm in diameter. The lower part of their culms usually has numerous branches forming a more or less dense, interwoven structure equipped with many sharp, hard thorns. The thorns of this bamboo group originate from shortened branch shoots (Arber 1965).

*Bambusa* has been divided into four subgenera—*Bambusa* subg. *Bambusa*, *Bambusa* subg. *Dendrocalamopsis* L.C.Chia & H.L.Fung, *Bambusa* subg. *Leleba* (Rumphius ex Nakai) P.C.Keng ex L.C.Chia & H.L.Fung, and *Bambusa* subg. *Lingnania* (McClure) L.C.Chia & H.L.Fung (Xia et al. 2006; Shi et al. 2021)—based on morphological traits such as culm sheath parts (comprising blade, auricles, and apex), culm internodes, and branchlets of lower branches.

Species delimitation within *B.* subg. *Bambusa* remains unsatisfactory and unresolved, posing a major challenge for taxonomy (Wang et al. 2022). This difficulty arises from several issues. Traditional and rigorous identifications rely primarily on morphological traits (Stapleton 1997). However, vegetative characteristics are often influenced by environmental factors such as habitat, climate, and soil composition, which are the main causes of morphological variation (Liu et al. 2024). Moreover, the flowering interval is long and uncertain (Janzen 1976), resulting in a scarcity of floral material for taxonomic study (Qin et al. 2022). Consequently, there is insufficient evidence to confirm the identification of specimens of these taxa, especially at the species level, without support from molecular data (Haevermans et al. 2020). However, in molecular phylogenetic studies, *Bambusa* Schreb. is not a monophyletic genus, as its species cluster together with *Dendrocalamus* and *Gigantochloa* species, which constitute the DBG complex group of paleotropical woody bamboos. These issues may be due to hybridization events between species in different genera or unresolved lineages (Goh et al. 2013; Liu et al. 2020).

This work focuses on thorny bamboos. Although they are difficult to identify at the species level, especially during their vegetative stage, thorny bamboos nevertheless have considerable value in many areas. They can be used as woody materials for construction due to their load-bearing capacity and for handicrafts owing to their flexibility and durability (Mohan et al. 2022), or for erosion control and dike protection when planted as hedges along riverbanks (Sungkaew and Teerawatananon 2017). In addition, several studies have revealed their potential for CO_2_ absorption, owing to their high biomass and sustainable growth (Diep et al. 2024).

However, the concept of “thorny bamboos” remains ambiguous and needs clarification before listing the species concerned. The main uncertainty lies in determining which species should be considered truly thorny bamboos based on the thorns on their branches: do they have hard, sharp thorns, blunt thorns, soft thorns, weak thorns, or a combination of these different thorns on the same branch? In many early studies, thorny bamboos were listed together with other *Bambusa* species in general bamboo checklists, without comprehensive or separate treatment (Munro 1868; Camus 1913). However, it is possible to identify thorny bamboos in these works because the authors often recorded certain species as having hard, sharp thorns on their branches. Recently, two large-scale studies on bamboos in China have addressed numerous species with hard, sharp, or soft thorns on their branches. Although these studies have not established a separate group for thorny bamboos, they considered thorns a major trait and placed all species bearing the above branch characteristics in *B.* subg. *Bambusa* (Xia et al. 2006; Shi et al. 2021).

No identification key for thorny bamboos in Asia exists. To address this gap, an online multi-access identification key will be created using the Xper^3^ platform. This platform allows users to work remotely on the same database simultaneously, thereby supporting collaborative taxonomic work and providing an effective tool for standardizing taxonomic descriptions. Accordingly, this study aims to (1) compile a comprehensive checklist and knowledge base of thorny bamboo species in Asia by thoroughly reviewing the relevant literature and collection specimens and (2) develop a practical tool for field identification and characterization of thorny bamboo species in Asia.

## Materials and methods

### Literature and collection specimens

Information on taxa related to thorny bamboos in Asia and their morphological descriptions was collected from the literature (see Table 1) and from observations of specimens, particularly nomenclatural types, at the Paris Herbarium (P). Digitized type vouchers and collection specimens from various other herbaria accessible via the Global Plants website JSTOR (2025) were also examined. Additional reference websites consulted in this study include BHL (2025), GBIF (2025), POWO (2025), WFO (2025), and Smithsonian Learning Lab (2025).

**Table 1.** A checklist of native thorny bamboos in Asia, including information on their native distribution, the publications used for extracting morphological traits, and the collection specimens examined in this study.

No.	Species	Native distribution (POWO 2025)	Publications used for extracting morphological traits	Additional materials examined in this study
1	*Bambusa angustissima* L.C.Chia & H.L.Fung	China (Guangdong)	Chia and Fung (1981); Xia et al. (2006); Shi et al. (2021); POWO (2025)	
2	*Bambusa bambos* (L.) Voss	Indian Subcontinent to Indochina	Gamble (1896); Camus (1913); Soderstrom and Ellis (1988); Pham (1993); Wong (1993); Dransfield and Widjaja (1995); Seethalakshmi et al. (1998); Lin (2000); Nguyen (2006); Shi et al. (2021); POWO (2025)	*T. R. Soderstrom & S. Kulatunge 1762* (US00057961) image!
				*T.R. Soderstrom 2608*
				(US00018878) image!
				*T. R. Soderstrom 2082*
				(P02326430) image!
				*M.H. Diep, J. Gurgand, N.N. Vien, C. Loeung & O. Sichaleune 508*
				(P02280554) image!
3	*Bambusa spinosa* Roxb.	Indonesia to Philippines (Jawa to Maluku)	Gamble (1896); Camus (1913); Pham (1993); Wong (1993); Dransfield and Widjaja (1995); Nguyen (2006); Xia et al. (2006); Shi et al. (2021); POWO (2025)	*Merrill, E.D*
				(K000290513) isotype image!
				*Zollinger 3417*
				(P02325986) image!
				*M.H. Diep, J. Gurgand, T.L. Cao, V.P. Le, N.G. Cao et T. Bui 112*
				(PBB000557) image!
4	*Bambusa chunii* L.C Chia & H.L.Fung	Laos	Chia et al. (1983); But et al. (1985); Xia et al. (2006); Shi et al. (2021); POWO (2025)	*Nan-zhu 2802*
				(K000854770) isotype image!
5	*Bambusa dissimulator* McClure	China (Guangdong) to Vietnam	Pham (1993); Xia et al. (2006); Shi et al. (2021); POWO (2025)	*H. Fung BG-2348*
				(P00633805) type collection image!
				*F. A. McClure A-674*
				(US00130312) holotype image!
				*F. A. McClure 20726*
				(US00032198) image!
6	*Bambusa flexuosa* Munro	China (Guangdong) to Indochina	Camus (1913); But et al. (1985); Pham (1993); Nguyen (2006); Xia et al. (2006); Shi et al. (2021); POWO (2025)	*Hance 1805*
				(P00633806) isotype image!
				*F.A. McClure 20674*
				(US00032223) image!
				*F.A. McClure 20737*
				(US00032226) image!
				*F.A. McClure 20737*
				(US00032227) image!
				*H. Fung 21208*
				(US00034836) image!
7	*Bambusa funghomii* McClure	China (Henan, Guangdong, Guangxi)	Xia et al. (2006); Shi et al. (2021); POWO (2025)	*F.A. McClure 20717*
				(US00130324) holotype image!
				*F.A. McClure 20717*
				(US00130323) image!
8	*Bambusa glabrovagina* G.A.Fu	China (Hainan)	Fu (1982); Xia et al. (2006); Shi et al. (2021); POWO (2025)	
9	*Bambusa lapidea* McClure	South China	But et al. (1985); Xia et al. (2006); Shi et al. (2021); POWO (2025)	*H. Fung 1114*
				(US00130328) holotype image!
				*H. Fung 21291*
				(P03234478) image!
				*Nan Zhu 2759*
				(K002875941) image!
				*F.A. McClure 21291*
				(P03224478) image!
				*F.A. McClure 00655*
				(US00034887) image!
10	*Bambusa latideltata* W.T.Lin	China (Guangdong)	Xia et al. (2006); Shi et al. (2021); POWO (2025)	
11	*Bambusa malingensis* McClure	China (Hainan)	But et al. (1985); Xia et al. (2006); Shi et al. (2021)	*H. Fung 20986*
				(US00130330) type image!
				*H. Fung 20986*
				(US00289558) from type plant image!
				*Nan Zhu 2765*
				(K002875947) image!
12	*Bambusa rutila* McClure	South China	But et al. (1985); Xia et al. (2006); Shi et al. (2021); POWO (2025)	*F. A. McClure 20683-A*
				(K000854752) holotype image!
				*F. A. McClure 1476*
				(US804004) image! Original material
				*P. F. Li 160*
				(P00697301) from living type image!
13	*Bambusa sinospinosa* McClure	South China to Hainan	But et al. (1985); Nguyen (2006); Xia et al. (2006); Shi et al. (2021); POWO (2025)	*H. Fung 20773*
				(US00130342) holotype image!
				*H. Fung 20773*
				(US00130343) holotype image!
				*F. A. McClure 20685*
				(US00034974) image!

### Software platform: Xper^3^

To produce the database and publish an online interactive identification key, we used Xper^3^. This software platform is a data management system dedicated to taxonomic descriptions (Vignes Lebbe et al. 2016; Kerner et al. 2025). Its main interface includes several tabs as follows: an “Items” tab allows the input of information on taxa; a “Descriptive model” tab defines unified terminology as a list of descriptors (characters) and descriptor states (character states); the “Description” tab connects the “Items,” “Descriptors,” and “Descriptor states” tabs as a matrix; and finally, the “Identification” tab provides access to an interactive identification key or allows computation of printable single-access keys. For more information, the Xper^3^ homepage can be accessed at https://www.xper3.fr/, which includes links to online documentation.

### Illustrations

Almost all images used to illustrate the descriptors and descriptor states on Xper^3^ were taken during field trips, mainly at Phu An Bamboo Village in Vietnam (https://ecobambou-phuan.org/). Additional images were obtained from voucher specimens in the Herbarium Paris (P), the Herbarium Phu An (PBB), and other virtual herbaria. When needed, original drawings were also produced. Field trip images were mainly captured with a Canon EOS 800D using a standard Canon EF-S 18–55 mm f/4–5.6 lens or a macro Canon EF 100 mm f/2.8 lens. Images of small-sized characters, including floral and leaf components observed under a microscope, were taken using an iPhone 14 Pro Max or other common smartphones.

### Defining the checklist of Asian thorny bamboos

According to the definition of thorn hardness by Pérez-Harguindeguy et al. (2013), thorns are considered soft if they can be easily bent when pressed by a finger at maturity, or hard if they cannot be bent under the same pressure. In this study, we restricted thorny bamboos to species possessing at least hard, sharp thorns on their branches (sometimes bearing both hard, sharp, and soft thorns simultaneously).

First, we reviewed relevant literature to determine whether the descriptions mentioned branches with hard, sharp thorns (see Suppl. material 1). We then examined herbarium specimens, including type specimens when available, to ensure consistency between the literature descriptions and the observed morphological traits of branches.

### Creating a descriptive database using software Xper^3^

We followed the instructions provided on the official Xper^3^ website (https://www.xper3.fr/) and in a recent guide by Kerner et al. (2025). First, we defined characters and character states from the literature and compiled species descriptions. These data were then entered into the Xper^3^ database.

A list of vegetative characters and character states initially compiled by Diep et al. (2016) was used as a starting point for our character list. We refined these to better represent the descriptions of Asian thorny bamboos found in the literature. Diagnostic floral characters were then added to further distinguish species and enrich species descriptions with as many morphological traits as possible.

Botanical terminology in our database was standardized to follow McClure (1966), Beentje (2010), and Diep et al. (2016). The final list of characters and character states (“descriptors” and “descriptor states” in Xper^3^) is provided in Suppl. material 2.

**Figure 1.**
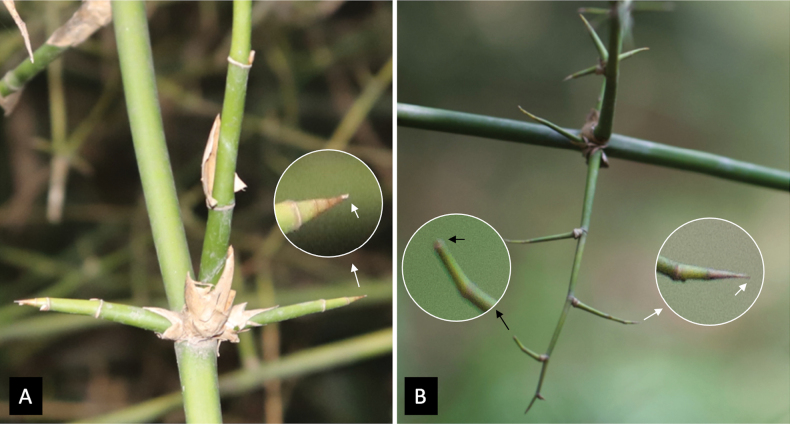
Illustration of the descriptor “Characteristics of thorns on branches” and the two character states: A. “Hard, sharp thorns only”; B. “Soft and hard thorns on the same branch.” These photos were taken at Phu An Bamboo Village by Nguyen Khac Dieu (A – *Bambusa sinospinosa* McClure) and Nguyen Phuoc Dan (B – *Bambusa* sp., Vietnamese vernacular name: Tre nga (Thai Nguyen)).

All selected species (the “item” concept in Xper^3^) were described according to the model (descriptors and states) established in the previous step. We extracted data from various publications to capture morphological variation among specimens from different geographic areas. Illustrative images were added, along with short explanatory texts on botanical terms when necessary (e.g., Fig. 1).

Once the database was created, various Xper^3^ tools were employed to verify its completeness and consistency, such as “Checkbase.” This tool includes item checking (to verify whether all species are distinct), state checking (to detect unused states and states that are always present), and description checking (to identify errors related to inapplicable descriptor rules).

### Interactive identification key

The tool Mkey (Kerner et al. 2025) was then used to display an online interactive identification key. At each identification step, the algorithm sorts the characters according to their efficiency in distinguishing the remaining species; however, users can be encouraged to select certain characters by assigning different weights to them. To determine which characters are more important than others, we based the weighting on the classical identification key of Xia et al. (2006).

## Results

### The checklist of thorny bamboos in Asia

The identification of bamboo species bearing only hard, sharp thorns or both hard, sharp, and soft thorns on their branches—based on literature sources and herbarium collections—resulted in a final list of 13 thorny bamboo species in Asia, presented in Table 1. Infraspecific taxa were excluded due to limited documentation. The interpretation supporting this list is provided in Suppl. material 1 and further discussed in the Discussion section.

Our data show that almost all thorny bamboos in Asia are native to China, with 10 out of 13 species distributed in tropical to subtropical climate regions. Other species in China, such as *B. dissimulator* McClure and *B. flexuosa* Munro, share their natural distribution with Vietnam and Indochina, respectively. Meanwhile, *B. bambos* (L.) Voss naturally occurs from India to Indochina, and *B. spinosa* Roxb. from Indonesia to the Philippines, while *B. chunii* L.C.Chia & H.L.Fung is restricted to Laos.

### Database on Xper^3^

#### The descriptors and the descriptor states

Morphological descriptors were extracted from the publications listed in Table 1 and are clearly presented in Suppl. material 2.

A total of 165 descriptors were established, comprising 108 vegetative and 57 reproductive descriptors, corresponding to 447 descriptor states. These descriptors were organized into 12 descriptor groups (see Suppl. material 2).

The characters and their corresponding weights are summarized in Table 2. Among them, the “Characteristics of thorns on branches” and “Presence of culm sheath auricles” were assigned the highest weight values to reflect their primary taxonomic importance. Following these, 18 additional characters were assigned a weight of 4. All remaining characters were given the default weight of 3, as they are considered to have comparable discriminatory value.

**Table 2.** Characters and their weights. The weights define a priority order in the list of descriptors in the key. Xper^3^ allows a weight ranging from 1 (lowest priority) to 5 (maximum priority).

No.	Characters	Weight
1	Characteristics of thorns on branches	5
2	Culm sheath auricles presence	5
3	Culm sheath auricles conspicuous or not	4
4	Culm sheath auricles equal to subequal or unequal	4
5	Culm sheath outer surface with hairs	4
6	Distribution of hairs on culm sheath outer surface when it is not uniform	4
7	Density of hairs on culm sheath outer surface	4
8	Culm internode surface with hairs	4
9	Culm sheath apex shape	4
10	Culm sheath blade base width to culm sheath apex width ratio	4
11	Culm sheath ligule tall	4
12	Nodal line with hairs above and below	4
13	Culm sheath shoulders shape	4
14	Culm sheath auricles shape	4
15	The large auricle width to the smaller auricle width ratio	4
16	Culm sheath auricles surface	4
17	Culm internode surface at base with stripes	4
18	Leaf blade adaxial surfaces	4
19	Leaf blade abaxial surfaces	4
20	Culm tall	4
21	145 remaining characters	3 (default)

#### Checkbase

A total of 956 cells (out of 2,145) in the Xper^3^ data matrix are unknown values, representing 44.6% of the 13 species × 165 descriptors. These unknown cells reflect the incomplete nature of morphological descriptions of thorny bamboos in the literature and inconsistency among authors in providing a standardized list of common characters and character states necessary for species descriptions. This missing information may be completed and refined in future studies.

### Completed identification key (multi-access key)

**Figure 2.**
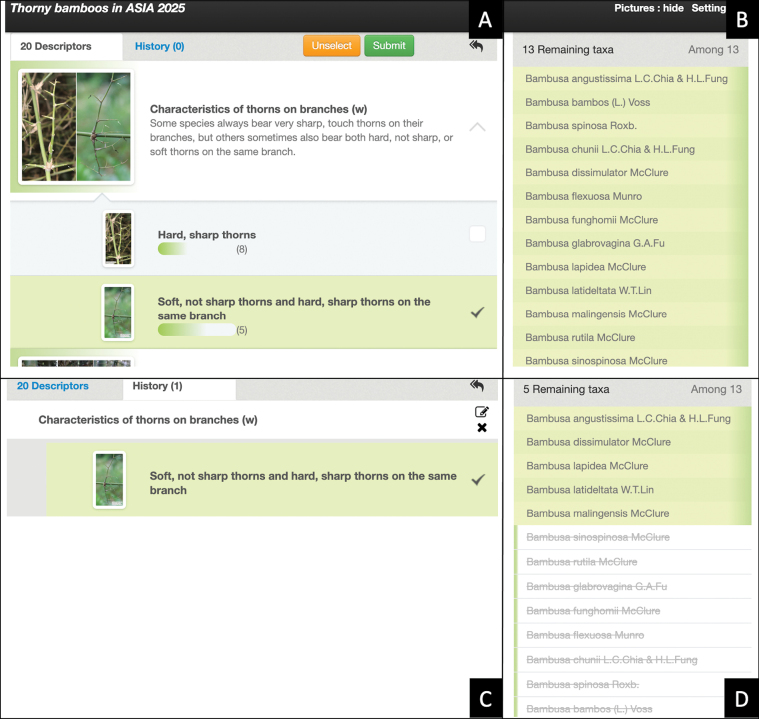
Example of the interface of the key. A. Descriptors and descriptor states with illustrative images and definitions; B. Taxa before the identification process; C. Description of the specimen to identify, for one descriptor; D. Results after describing the specimen for the characteristics of thorns on branches: five taxa retained, others discarded.

The interface of the key (Fig. 2) includes the descriptors (Fig. 2A) on the left side and taxa (Fig. 2B) on the right side. Users are free to select the descriptors they wish to observe to identify a specimen. The descriptors are automatically sorted according to their weights (see Table 2). When weights are equal, the order is based on their ability to differentiate between species. During the identification process, when a descriptor is defined for the specimen to be identified (Fig. 2C), one or more species are discarded, while others are retained (Fig. 2D). The process stops when the user chooses to interrupt it or when only one species remains on the right side.

### Species profiles

**Figure 3.**
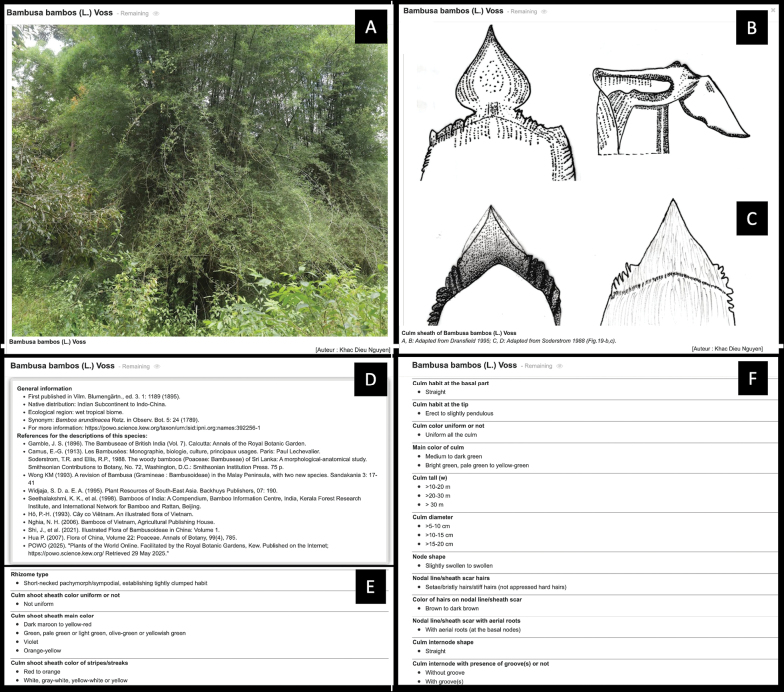
Screenshots showing part of the species profile of *B. bambos* (L.) Voss as an example. A. Image showing clumps with dense clusters of branches at the base; B, C. drawings of the culm sheath adapted from Dransfield and Widjaja (1995) and Soderstrom and Ellis (1988), respectively; D. general information of the species; E, F. part of the detailed description of the species.

Users can access the profiles of the 13 species before or after interacting with the key by clicking on the names of the taxa. For example, the profile of *B. bambos* (L.) Voss is shown in Fig. 3. Each species profile includes images of its diagnostic traits and general information, such as the first publication of the species, synonyms, references describing the species, and a detailed description using descriptors and descriptor states from the Xper^3^ data matrix. Although the database contains a total of 165 characters, the characters appearing in the species profiles are generally fewer due to the inapplicability of some descriptors (e.g., auricle shapes in the absence of auricles) and unknown data.

**Figure 4.**
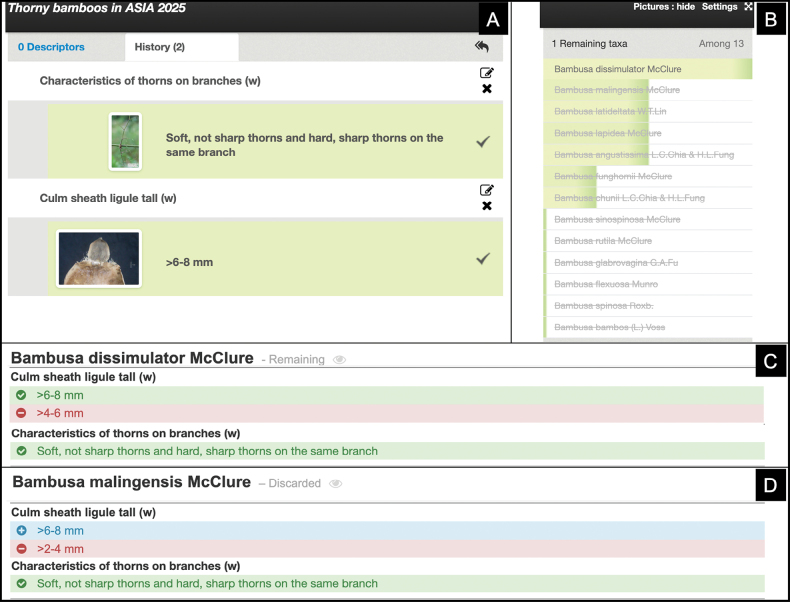
Example of the identification process. A. The user selected “soft, not sharp thorns and hard, sharp thorns on the same branch” for the first step of the key and then “>6–8 mm” for the culm sheath ligule; B. only one species in the list is fully compatible—*B. dissimulator*; C. in the *B. dissimulator* species profile, the two descriptors (“Culm sheath ligule tall” and “Characteristics of thorns on branches”) are compatible with the states selected in (A), as indicated by the green coloring; D. conversely, *B. malingensis* has a short culm sheath ligule (2–4 mm), never exceeding 6–8 mm. Therefore, this species is not compatible with the specimen, which has been identified as *B. dissimulator*.

At any point during the identification process, different colors are used in the species profiles to explain why the specimen has been identified as this species or not (Fig. 4). Green indicates states compatible with the specimen being identified, blue indicates states selected for the specimen that justify the elimination of the species, and red indicates states possible for the species but not selected during identification.

## Discussion

### Literature analysis and species checklist

The list of 13 species of thorny bamboos in Asia was compiled through careful examination of various published sources, with detailed information provided in Suppl. material 1.

The earliest publication on bamboos in general—and thorny bamboos in particular—was that of Linnaeus (1753), which included only *Arundo bambos* L., now *B. bambos* (L.) Voss, with a very brief description and a note on its habitat in India. This species has been reported to bear thorns on its branches (Xia and Stapleton 1997). Munro (1868) subsequently described several bamboo species with *culmus spinosus*, including *B. arundinacea* (Retz.) Willd., *B. orientalis* Nees, *B. blumeana* Schultes, *B. spinosa* Roxb., and *B. flexuosa* Munro. The first two species and the latter two are now considered synonyms. Thus, the number of thorny bamboos recorded by Munro (1868) is currently reduced to three species: *B. arundinacea* (Retz.) Willd., *B. spinosa* Roxb., and *B. flexuosa* Munro. According to POWO (2025), *B. arundinacea* (Retz.) Willd. is treated as a synonym of *B. bambos* (L.) Voss. While *B. spinosa* Roxb. is officially recognized, its synonym *B. blumeana* Schultes is still widely used by researchers. Subsequently, Gamble (1896), Camus (1913), and Arber (1965) described and mentioned several species having thorns on their branches. In the early stage of bamboo classification, no thorny bamboo species bearing soft thorns had been recorded. In other words, nearly all species discussed above represent thorny bamboos with hard, sharp thorns, except for *B. dissimulator* McClure, which was reported by McClure (1966) to have less obvious thorns.

But et al. (1985), in their work on the bamboos of Hong Kong, described nine species with branches bearing hard or soft thorns. Wong (1993) and Dransfield and Widjaja (1995) mentioned only two thorny bamboos with hard, sharp thorns on their branches, *B. bambos* and *B. blumeana*, in Southeast Asia. Based on brief descriptions in the “Checklist of Bamboos of the World” by Ohrnberger (1999), eight species were considered thorny bamboos. Pham (1993) and Nguyen (2006), in their studies on the “Flora of Vietnam” and “The Bamboos of Vietnam”, respectively, mentioned about four species of thorny bamboos in Vietnam. Franklin (2003) estimated that there were about 15 species of thorny bamboos in *Bambusa*.

More recently, Xia et al. (2006) listed 27 species with branches bearing hard or soft thorns, while Shi et al. (2021) reported 28 species in the “Illustrated Flora of Bambusoideae in China”. Qin et al. (2022) provided the first detailed description of floral morphology and designated epitypes for four Chinese *Bambusa* species, accompanied by high-quality illustrative images. Among these, *B. corniculata* L.C.Chia & H.L.Fung and *B. cornigera* McClure were reported to bear weak thorns. During this period, the terminology “soft thorns” or “weak thorns” on bamboo branches was usually applied to characterize several species of *B.* subg. *Bambusa* in China. In contrast, thorny bamboos in Southeast Asia were not reported as bearing soft thorns.

The 28 species having hard, sharp, or soft thorns listed in Suppl. material 1 were selected from the 35 species of *B.* subg. *Bambusa* (Shi et al. 2021) for discussion, as they represent almost all bamboos with thorn-bearing branches that have been recorded. Following the descriptions of the above authors and considering the species matching the criteria outlined in the Methods section, the final list was established. However, *B. gibba* is an exception to the criteria applied. Although Ohrnberger (1999) treated it as a thorny bamboo, Pham (1993) and Nguyen (2006) reported this species in Vietnam as having no thorns, and its vernacular names, “Tre bầu” and “Luồng may,” do not suggest an association with thorny bamboos. Further examination of type specimens provided no evidence that this species is a thorny bamboo.

### Database

**Figure 5.**
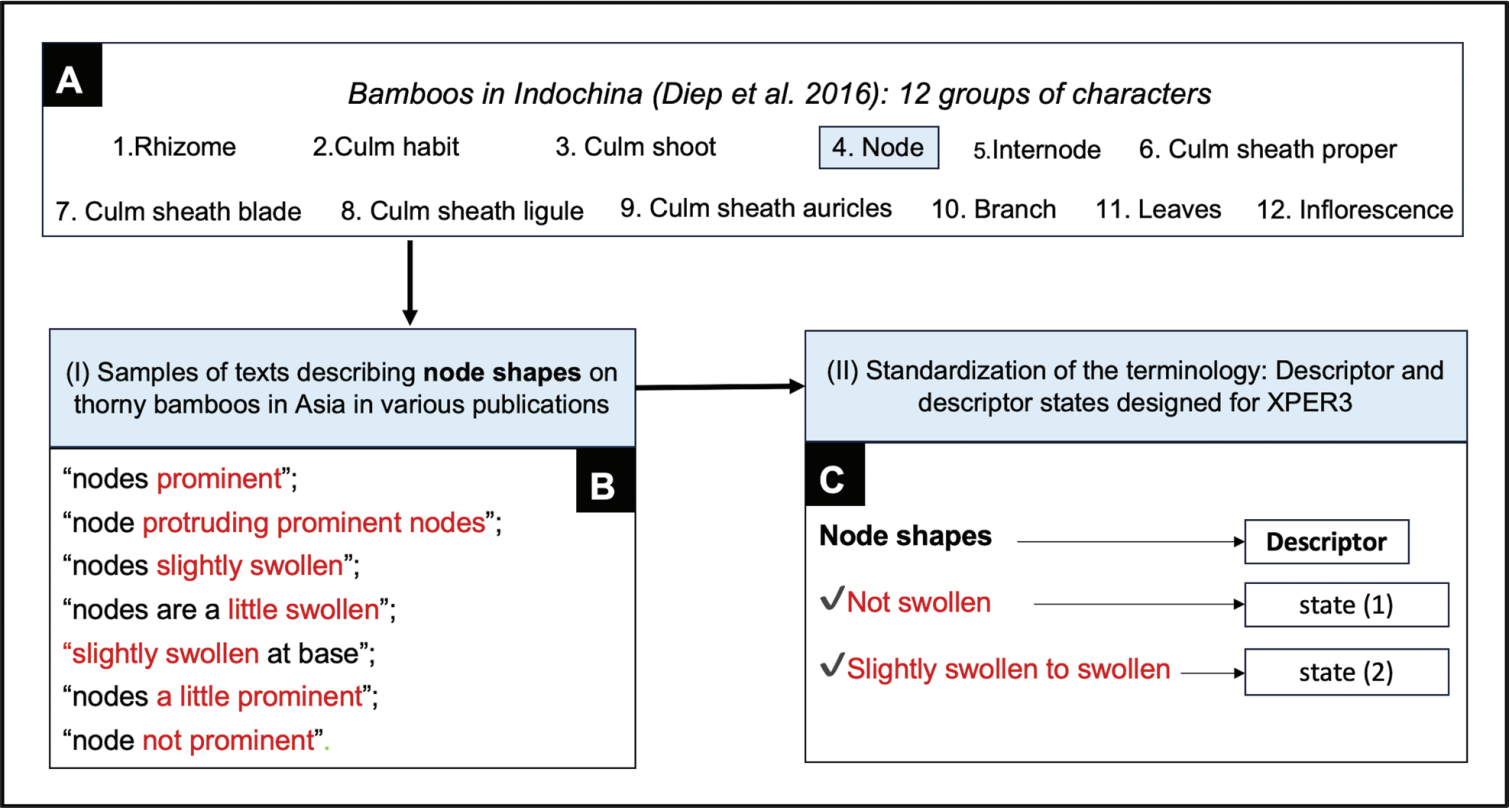
Example of the workflow used for databasing morphological characteristics of thorny bamboos in Asia from the literature. A. Previous terminology defined by Diep et al. (2016), with characters sorted into 12 groups related to different vegetative parts; B. analysis of publications describing thorny bamboos; C. choice of terminology for databasing, focusing on the main objective—here, “swollen.”

Botanical terminology used in specimen descriptions is often inconsistent or ambiguous among authors, as noted by Tyyskä and Atkins (2025). Terms used for thorny bamboos in Asia are no exception. Several descriptive terms—particularly those referring to shape—have been applied differently by various sources, making interpretation difficult. For instance, as illustrated in Fig. 5, terms such as “prominent” and “swollen” are often used interchangeably. In this study, we chose to retain “*swollen*” as the preferred term and to treat “*prominent*” as its synonym. Likewise, the modifier “*slightly*” was considered too subjective, and we merged “slightly swollen” and “swollen” into a single descriptor state, “*swollen*”, to ensure clarity and consistency in the dataset.

Another issue is the lack of information on some key characters when databasing species on Xper^3^, especially those related to shoot characters, which are regarded as diagnostic traits by several authors. Recent publications on new species of bamboos have included these characters (Wong and Diep 2015; Tran 2021; Arthan et al. 2023; Ni et al. 2025). Diep et al. (2016) also created a database based on 12 groups of descriptors that included culm shoot characters for bamboos in Indochina. However, in the recent literature on thorny bamboos, shoot characteristics are often not described; instead, the sheath of the young culm is mentioned. Therefore, we used the characteristics of the young culm sheath described in the literature to represent the culm shoot sheath (see characters No. 2–4 in Suppl. material 2).

Given the completeness of the database, some noteworthy information about thorny bamboos can be extracted through analysis. The “Comparison” function in Xper^3^ highlights common or possible characteristics for the 13 species. These characteristics can be compared between *B.* subg. *Bambusa*, as defined by Xia et al. (2006), and the 13 species in our checklist (see Table 3). It appears that the Xper^3^ database encompasses a broader range of morphological variability. This is due to the variety of materials we used, including specimens and publications describing the species from a wider geographical range than those used in descriptions of *B.* subg. *Bambusa* species in China.

**Table 3.** Comparison of descriptors between *B.* subg. *Bambusa* (as defined by Xia et al. (2006)) and the thorny bamboos in Xper^3^ (as extracted from the union characteristics in Xper^3^).

No.	Feature characters	*B.* subg. *Bambusa* (Xia et al. 2006)	Thorny bamboos in Asia from Xper^3^
1	Culm internode length	< 30 cm	4–50 cm
2	Culm wall thick	up to 2 cm	<1–(2)–5 cm
3	Culm sheaths	thickly leathery	thick/leathery/coriaceous
4	Culm sheath auricles	auricles large, rounded or irregular, or absent;	auricles present or absent;
			size: 2–10 mm large, less than 50 mm long;
			shape: various shapes from linear to crescent, ovate, lanceolate, oblong to crescent, and lobe-like or undulate.
5	Culm sheath blade	blade persistent, broad, base 1/2–3/4 width of sheath apex.	blade persistent;
			shape: triangular, lanceolate, or ovate;
			base 1/3 to 2/3 width of culm sheath apex.
6	Branch position on culms	arising from basal, mid-culm, and apical nodes	arising from all culm nodes (except basal ones), or arising from all culm nodes
7	Branch characteristics	usually 3 co-dominant;	solitary branch, 2 branches with 1 primary branch and 1 smaller, or 3 branches to several
8	Branchlet characteristics	branchlets of lower branches specialized into tough or weak thorns	branches have soft, not sharp thorns or hard, sharp thorns
9	Pseudospikelets	loose at maturity, with broad florets on short rachilla segments.	rachilla < 4 mm

### Interactive identification key

Electronic identification keys, especially those using Xper^3^ software, have become increasingly popular in recent years, as demonstrated by several studies (Lombard et al. 2021; Tyyskä and Atkins 2025; Särkinen et al. 2025). These studies highlight the significant contribution of information technology to modern plant taxonomy.

The present study publishes a unique key that promises potential application for future bamboo taxonomy research. This key can serve as a practical pocket tool for quickly identifying Asian thorny bamboos in the field. By taking detailed steps to establish a database and extract morphological characters from diverse materials, this key covers as many variations of thorny bamboos as possible. It may also inspire further work on the entire *Bambusa* genus or related genera.

Furthermore, the key offers several advantages. First, the construction of the key is not fixed, as information can be easily updated in the original database to improve its performance and accuracy. For example, morphological characters of shoots or flowers are often absent in living specimens because shoots grow seasonally, while the flowering interval is long and unpredictable. These can be added when available. Second, the Xper^3^ platform facilitates sharing, collaboration, and feedback from colleagues and users. When new traits are observed in a species by others, they can be readily incorporated into the dataset, ensuring that the key is continually updated. Third, users can begin the identification process at any character or navigate the steps according to the materials they have or the features they prefer to examine. Finally, the image illustrations for each character make the key intuitive and easy to use, as the descriptions and images are displayed together, unlike in traditional keys. These visual features help non-specialist users better understand and perform the identification steps.

In this key, we have already included floral characters in the database, together with fresh illustrative images—such as anthers, filaments, stigmas, styles, ovaries, and lodicules—which meaningfully contribute to enhancing the understanding of the reproductive structures of thorny bamboos, as these are usually examined only in dry herbarium specimens or old drawings.

### Species profiles

The species profiles serve as practical companions to the interactive key, offering visual and descriptive references for each thorny bamboo taxon. Each profile includes diagnostic notes, photographs, known distribution data, and protologue references to help confirm species identity. The photographs show the general habit of the culm, culm sheath, and pseudospikelets, which aid users in gaining a complete understanding of each species. Moreover, the species profiles are presented within the same interface as the interactive identification key. This integration is useful because, at the end of the identification process, users can immediately access detailed information on the characters used to differentiate each species from others.

## Conclusion

The development of a morphological database and interactive key for 13 Asian thorny bamboo species establishes a practical foundation for future taxonomic research on this group. By integrating illustrations with clearly defined descriptors, the key enhances the reliability of species-level identification. Preliminary validation with herbarium material from MNHN indicates internal consistency, although further assessment by the wider botanical community will be essential for refinement. The study also highlights notable gaps in descriptive information, which constrain the completeness of the database. Continued fieldwork and detailed morphological study remain critical not only for updating and expanding this resource but also for advancing a more comprehensive understanding of Asian thorny bamboos.

## Additional information

### Use of AI

No AI tools were used to generate the manuscript. DeepL and ChatGPT-5 were occasionally used to check and improve grammar or translations.

### Author ORCIDs

My Hanh Diep https://orcid.org/0009-0001-0870-9677

Marc Pignal https://orcid.org/0000-0002-6772-9299

Régine Vignes Lebbe https://orcid.org/0000-0002-6912-6248

### Data availability

All of the data that support the findings of this study are available in the main text or Supplementary Information.

## Supplementary materials

Supplementary material 1 The list of bamboos in Asia having soft thorns and hard thorns on their branches Data type xlsx Explanation note The matrix reports the characteristics of thorns described in the publications and additional collection specimens used for examination. In the last column on the right side we discarded the species bearing only soft thorns and kept 13 species for this study. https://binary.pensoft.net/file/1526748This dataset is made available under the Open Database License (http://opendatacommons.org/licenses/odbl/1.0/). The Open Database License (ODbL) is a license agreement intended to allow users to freely share, modify, and use this Dataset while maintaining this same freedom for others, provided that the original source and author(s) are credited. Khac Dieu Nguyen, My Hanh Diep, Marc Pignal, Régine Vignes Lebbe

Supplementary material 2 List of morphological characters and character states of thorny bamboos in Asia Data type xlsx https://binary.pensoft.net/file/1526749This dataset is made available under the Open Database License (http://opendatacommons.org/licenses/odbl/1.0/). The Open Database License (ODbL) is a license agreement intended to allow users to freely share, modify, and use this Dataset while maintaining this same freedom for others, provided that the original source and author(s) are credited. Khac Dieu Nguyen, My Hanh Diep, Marc Pignal, Régine Vignes Lebbe
